# Exosomal miRNA-148b/301a/423 cluster predicts pneumonitis risk in NSCLC with concurrent radiotherapy with immunotherapy via PTPN14-YAP signaling: a retrospective cohort study

**DOI:** 10.3389/fimmu.2025.1666946

**Published:** 2025-09-26

**Authors:** Jiaming Cao, Jiaqi Zhang, Wenbo Zhao, Baosen Zhou, Chang Zheng

**Affiliations:** ^1^ Department of Clinical Epidemiology and Evidence-based Medicine, The First Hospital of China Medical University, Shenyang, China; ^2^ Electrodiagnosis Department, Shenyang Fifth People Hospital, Shenyang, China; ^3^ Medical Laboratory Technology, School of Medicine, Hebei University of Engineering, Handan, China

**Keywords:** non-small cell lung cancer, radiotherapy, immunotherapy, pneumonitis, miRNA cluster

## Abstract

**Background:**

Radiotherapy (RT) combined with Immune checkpoint inhibitors (ICIs) significantly improve outcomes in non-small cell lung cancer (NSCLC), yet this combination amplifies treatment-related pneumonitis risk. The real-world incidence, predictive biomarkers, and underlying pathogenesis of RT-ICI-associated pneumonitis remain inadequately defined.

**Methods:**

We conducted a retrospective cohort study using electronic health records from 21,671 NSCLC patients treated with thoracic RT, categorized into RT-ICI (n=8,744) and RT-nonICI (n=12,927) groups after 1:1 propensity score matching. Pneumonitis was diagnosed via clinical/imaging criteria and infection exclusion. Incidence of pneumonitis was evaluated using propensity score–matched analysis, Kaplan-Meier curves, and Cox regression models. Subgroup analyses were performed across demographic and clinical variables. Serum exosomes from 20 patients underwent miRNA sequencing. LASSO regression for biomarker modeling, single-cell RNA-seq analysis and single-sample gene set enrichment analysis for function enrichment, then validated *in vitro*.

**Results:**

The incidence of pneumonitis was significantly higher in the RT-ICI group (28.9%) compared to the RT-nonICI group (10.0%) (hazard ratio [HR]=2.86; 95% confidence interval [CI], 2.43–3.29; P<0.001). This elevated risk persisted across age, sex, BMI, comorbidities, and autoimmune status. We identified a serum exosomal miRNA cluster (miR-148b-3p, miR-301a-3p, miR-423-3p) predictive of pneumonitis and poor survival. These miRNAs directly co-target PTPN14, and crosstalk with fibrosis via TNF signal at the single-cell level. Then we validated the miRNA cluster suppressed PTPN14, activating YAP signal to promote EMT in pulmonary epithelial cell lines.

**Conclusions:**

RT-ICI therapy significantly increases pneumonitis risk in NSCLC, especially in autoimmune comorbidities. A serum exosomal miRNA cluster (miR-148b-3p/301a-3p/423-3p) enables early pneumonitis prediction and prognosis assessment, offering novel targets for prevention and monitoring.

## 1 Introduction

Lung cancer remains one of the most commonly diagnosed malignancies worldwide and is the leading cause of cancer-related mortality, with an estimated 2 million new cases and 1.76 million deaths annually (1). Over the past decade, significant advancements have been achieved in the treatment of metastatic non-small cell lung cancer, leading to notable improvements in patient survival (2). In 2022, approximately 2.5 million new cases of lung cancer were reported globally, accounting for 12.4% of all new cancer diagnoses (3). Non-small cell lung cancer (NSCLC) constitutes approximately 85% of all lung cancer cases and is frequently diagnosed at an advanced stage, when curative interventions are often no longer feasible (4). Despite progress in chemotherapy and targeted therapies, the five-year survival rate for patients with metastatic NSCLC has historically remained below 7% (5).

In recent years, the combination of radiotherapy (RT) with immune checkpoint inhibitors (ICIs) has emerged as a promising strategy to enhance anti-tumor immunity and fundamentally reshape the therapeutic landscape of NSCLC (6). RT is postulated to augment systemic immune responses through mechanisms such as antigen release and T-cell priming, synergizing with ICIs to improve long-term outcomes (7). Clinical adoption of this combined approach has grown rapidly, with recent landmark trials demonstrating median overall survival exceeding 20 months in selected patients (8, 9).

However, the synergy between RT and ICIs may also amplify treatment-related toxicities. Pneumonitis-encompassing both radiation pneumonitis (RP) and checkpoint inhibitor-associated pneumonitis (CIP), represents a clinically significant complication of combined therapy (10). Patients with NSCLC frequently have pre-existing pulmonary comorbidities (e.g., COPD or interstitial lung disease), which may further increase susceptibility to lung injury during RT-ICI treatment (11). he reported incidence of treatment-related pneumonitis in RT-ICI cohorts ranges from 10% to 18%, with severe cases (Grade ≥3) accounting for up to 5% and contributing to treatment discontinuation or mortality (12). Key risk factors include thoracic radiation dose, fractionation schedules, ICI drug type, and baseline lung function, yet predictive biomarkers for early detection remain elusive (13).

Serum exosomes have recently emerged as promising vehicles for minimally invasive biomarker discovery, carrying molecular cargo (e.g., proteins, miRNAs) that reflect tissue-specific injury and immune activation (14). In the context of RT-ICI-associated pneumonitis, exosome signatures may provide critical insights into subclinical pathogenesis before symptomatic onset (15).

Despite increasing clinical recognition of RT-ICI-associated pneumonitis, large-scale studies integrating clinical outcomes with serum exosomes profiling are lacking. To address this gap, we conducted a matched cohort study to evaluate the incidence and risk factors of pneumonitis in NSCLC patients receiving combined RT-ICI therapy, then identify serum exosome-derived molecular signatures predictive of pneumonitis development and characterize inflammatory pathways driving treatment-related lung toxicity.

## 2 Materials and methods

### 2.1 Study design and population

We conducted a retrospective cohort study using electronic health records from a large tertiary class-A hospital in China, spanning from January 2010 to December 2023. Eligible participants included adults (≥18 years) with histologically confirmed NSCLC who initiated systemic anti-cancer therapy. Patients treated with definitive RT were stratified into two groups: those concurrent treated with ICIs and those receiving non-ICI regimens. Hospitalization days were calculated as from first anticancer treatment to last follow-up/death, excluded outpatient visits and emergency stays <24 hours. Patients with prior diagnoses of pneumonitis, interstitial lung disease, or incomplete clinical data were excluded from the study. This study was approved by the Ethics Committee of China Medical University (Approval No. [2022]175). Plasma from patients before RT-ICI therapy was obtained, collected in EDTA anticoagulation tubes, immediately centrifuged at 1,500 rpm for 10 min to separate plasma, then stored at -80°C until use. Following the ethical standards of the institutional and/or national research committee and the 1964 Helsinki Declaration and its subsequent amendments, this research was conducted with a waiver of informed consent, justified by the retrospective design and use of de-identified clinical data.

### 2.2 Exposure and outcome definitions

Radiotherapy exposure was defined as the administration of curative- or palliative-intent thoracic radiation with a cumulative dose ≥40 Gy as documented in medical records, while ICI exposure was defined as the receipt of PD-1, PD-L1, or CTLA-4 targeting agents approved by the FDA or NMPA per pharmacy administration records. The primary outcome of interest was RT-ICI-induced pneumonitis, which required fulfillment of diagnostic criteria including: 1) clinical symptoms (non-productive cough, dyspnea, and/or hypoxemia); 2) confirmatory CT findings demonstrating intra-radiation-field ground-glass opacities or consolidations; and 3) exclusion of infectious causes via microbiologic testing and imaging. Secondary endpoints included progression-free survival (PFS) and overall survival (OS).

### 2.3 Propensity score matching

To minimize baseline confounding, we performed 1:1 nearest-neighbor propensity score matching (PSM) using a caliper of 0.1 standard deviation. Covariates included in the matching model were age, gender, body mass index (BMI), comorbidities (hypertension, diabetes, ischemic heart disease, autoimmune disorders), smoking history, prior treatments, and healthcare utilization. After matching, covariate balance was assessed using standardized mean differences (SMD), where a value under 0.1 signified good balance.

### 2.4 Statistical analysis

The baseline demographics and clinical characteristics of the study population were summarized using descriptive statistics. Continuous and categorical variables were presented as mean ± standard deviation (SD) and frequency with percentage (%), respectively. Group comparisons were made using Student’s t-test for continuous variables and the chi-square test for categorical variables. Pneumonitis-free survival was visualized with Kaplan-Meier curves, and the log-rank test was used to evaluate differences. Finally, Cox proportional hazards models were utilized to determine hazard ratios (HR) and corresponding 95% confidence intervals (CIs). Three multivariable models were sequentially established with incremental adjustments to account for confounding factors: Model 1 was adjusted for age and sex; Model 2 further included adjustments for BMI and key comorbidities such as hypertension and diabetes; and Model 3 additionally accounted for immunosuppressant or antirheumatic medication usage. Subgroup analyses were conducted to investigate heterogeneity in pneumonitis risk across predefined variables, including sex, age categories, autoimmune disease status, and medication use. All statistical analyses were conducted using R software (version 4.2.1), and statistical significance was defined as a two-sided P-value <0.05.

### 2.5 Isolation, identification, and labeling of exosomes

The serum supernatant was processed using our standardized exosome isolation protocol (16): sequential centrifugation at 10,000×g (30 min, 4°C) and 100,000×g (70 min, 4°C), followed by PBS wash with repeat ultracentrifugation. Characterization included: (1) transmission electron microscopy (TEM) for cup-shaped morphology; (2) western blot for CD63/CD81/TSG101 biomarker validation; (3) nanoparticle tracking analysis (NTA, Zetasizer Lab, Britain) for size distribution.

### 2.6 Differential expression miRNA-seq analysis and target genes analysis

For biomarker insights into RT-ICI-induced pneumonitis, the serum samples from 10 patients in each group (RT-ICI vs. RT-nonICI) were collected and subjected to microarray analysis on an Illumina platform (BioMarker, China). The raw data were then normalized and examined in R with the “limma” package, followed by the identification of differentially expressed genes (DEGs) using a cutoff of |log2FC| > 1 and an adjusted P-value<0.05. The starBase database predicted the target genes of miRNAs. Luciferase activity was measured using the Dual-Luciferase^®^ Reporter Assay System (Promega) for validation as established protocols (17).

### 2.7 Single-cell seq analysis and pathway enrichment

Per our published pipeline (18), Enrichment analyses for Gene Ontology (GO) and the Kyoto Encyclopedia of Genes and Genomes (KEGG) were conducted using the R package “clusterProfiler” and Gene Set Variation Analysis (GSVA), respectively. Protein-protein interaction (PPI) network was constructed utilizing the GENEMANIA database; subsequently, single-cell RNA-seq data (GSE131907, LUAD) (19). underwent processing per established pipelines where Seurat v4 executed quality control followed by SCTransform normalization, PCA dimensionality reduction, and UMAP clustering, with cell-cell communication further deciphered by CellChat (ligand-receptor DB v1.1.0); finally, tumor microenvironment deconvolution was achieved via CIBERSORTx (LM22 signature, 1,000 permutations).

### 2.8 Expression validation and functional assays

For cell culture, BEAS-2B cells were cultured in DMEM supplemented with 10% FBS, while A549 cells were maintained in DMEM/F12K medium containing 10% FBS, both at 37°C with 5% CO_2_. Transfection was performed using JetPrime reagent following the manufacturer’s protocol, with medium replacement at 6h and assays conducted 24-48h post-transfection.

Gene expression was quantified via qRT-PCR using RNAiso Plus-extracted total RNA reverse transcribed to cDNA, U6/β-actin was utilized as the internal control. The transcription levels of the target genes were quantified using the 2^(-ΔΔCt) method. The sequences of the primers used were as follows: PTPN14-F 5’- CGACTTCTGGCAGATGGTGT -3’; PTPN14-R 5’- GTGGCTTTTGGTTCGTCCAC -3’; hsa-miR-148b-3p -F 5’- CGGTCAGTGCATCACAGAA -3’; hsa-miR-148b-3p - R 5’- GTGCAGGGTCCGAGGT -3’; hsa-miR-301a-3p -F 5’- GTATACCAGTGCAATAGTATT -3’; hsa-miR-301a-3p - R 5’- GTGCAGGGTCCGAGGT -3’; hsa-miR-423-3p -F 5’- ATAAGCTCGGTCTGAGGCCC -3’; hsa-miR-423-3p - R 5’- TATCCTTGTTCACGACTCCTTCAC -3’; U6-F 5’- GGAACGATACAGAGAAGATTAGC -3’; U6 - R 5’- TGGAACGCTTCACGAATTTGCG -3’; β-actin -F 5’- TCACCCACACTGTGCCCATCTACGA -3’; β-actin - R 5’- CAGCGGAACCGCTCATTGCCAATGG -3’.

For Western blot analysis, protein samples were lysed using RIPA buffer and separated by 10% SDS-PAGE. Subsequently, the proteins were transferred onto PVDF membranes. After blocking with 5% non-fat milk, the membranes were incubated overnight at 4°C with primary antibodies against PTPN14 (67744-1-Ig, Proteintech), YAP (66900-1-Ig, Proteintech), phosphorylated YAP S127 (p-YAP S127; ab76252, Abcam), E-cadherin (60335-1-Ig, Proteintech), and Vimentin (10366-1-AP, Proteintech). The membranes were then washed and incubated with HRP-conjugated secondary antibodies, and protein signals were detected using enhanced chemiluminescence.

Cell proliferation was assessed using CCK-8 assay by measuring absorbance at 450 nm at 0, 24, 48, and 72 hours post-seeding, performed according to our standardized protocols (20).

## 3 Results

### 3.1 Patient characteristics

Following 1:1 propensity score matching of 21,671 NSCLC patients, two balanced cohorts of 8,744 patients each were generated: one treated with RT and ICIs and the other with RT but non-ICI therapies. Demographic and clinical profiles, including age, sex, BMI, smoking history, comorbid conditions, and medication use, were comparable between the two groups, ensuring baseline equivalence (**Table 1**).

**Table 1:** Baseline characteristics of NSCLC patients with radiation therapy after propensity score matching, stratified by ICI treatment status.

Variable	RT-ICI (N=8744)	RT-nonICI (N=12927)	P	SMD
Age	59.56 ± 10.07	59.57 ± 10.07	0.959	0.001
Hospitalization Days	4.04 ± 2.81	4.04 ± 2.87	0.865	0.002
Sex				
Female	4346 (49.7)	6443 (49.8)	0.852	0.003
Male	4398 (50.3)	6484 (50.2)	0.852	0.003
ICI type				
PD-1	6961 (79.6)	—	—	—
PD-L1	1318 (15.1)	—	—	—
CTLA-4	465 (5.3)	—	—	—
Pneumonitis				
No	6221 (71.1)	11639 (90.0)	<0.001	0.478
Yes	2523 (28.9)	1288 (10.0)	<0.001	0.478
Death				
No	7446 (85.2)	11003 (85.1)	0.952	0.001
Yes	1298 (14.8)	1924 (14.9)	0.952	0.001
Hypertension				
No	6141 (70.2)	9123 (70.6)	0.599	0.007
Yes	2603 (29.8)	3804 (29.4)	0.599	0.007
Diabetes				
No	6997 (80.0)	10305 (79.7)	0.596	0.008
Yes	1747 (20.0)	2622 (20.3)	0.596	0.008
BMI Category				
18.5–24.9	3536 (40.4)	5139 (39.8)	0.596	0.014
25–29.9	3446 (39.4)	5205 (40.3)	0.596	0.017
<18.5	429 (4.9)	646 (5.0)	0.596	0.004
≥30	1333 (15.2)	1937 (15.0)	0.596	0.007
Tobacco Use				
No	7850 (89.8)	12519 (96.8)	<0.001	0.283
Yes	894 (10.2)	408 (3.2)	<0.001	0.283
Nicotine Dependence				
No	8019 (91.7)	12532 (96.9)	<0.001	0.226
Yes	725 (8.3)	395 (3.1)	<0.001	0.226
Antirheumatics				
No	7467 (85.4)	11872 (91.8)	<0.001	0.203
Yes	1277 (14.6)	1055 (8.2)	<0.001	0.203
Depressive Episode				
No	8115 (92.8)	12166 (94.1)	<0.001	0.053
Yes	629 (7.2)	761 (5.9)	<0.001	0.053
Rheumatoid Arthritis				
No	8580 (98.1)	12802 (99.0)	<0.001	0.077
Yes	164 (1.9)	125 (1.0)	<0.001	0.077

Continuous variables are presented as mean ± standard deviation (SD), and categorical variables are presented as frequency with corresponding percentage (%). Continuous variables include age (years) and hospitalization days (days). Categorical variables encompass demographic data (sex: male, female), ICI type (PD-1, PD-L1, CTLA-4; reported only for the RT-ICI group), clinical outcomes (pneumonitis: yes, no; death: yes, no), health-related behaviors (tobacco use, nicotine dependence), and comorbid conditions (hypertension, diabetes, BMI category [<18.5, 18.5–24.9, 25–29.9, ≥30 kg/m²], use of antirheumatic medications, depressive episodes, rheumatoid arthritis). Standardized mean differences (SMD) are presented as absolute values, where SMD values <0.1 indicate acceptable balance between the matched groups. Statistical significance (P-value) was determined using Student’s t-tests for continuous variables and chi-square tests for categorical variables. P values and SMDs are not applicable for RT-ICI type because RT-nonICI patients did not receive immune checkpoint inhibitors.

### 3.2 Incidence and timing of pneumonitis

**Figure 1:**
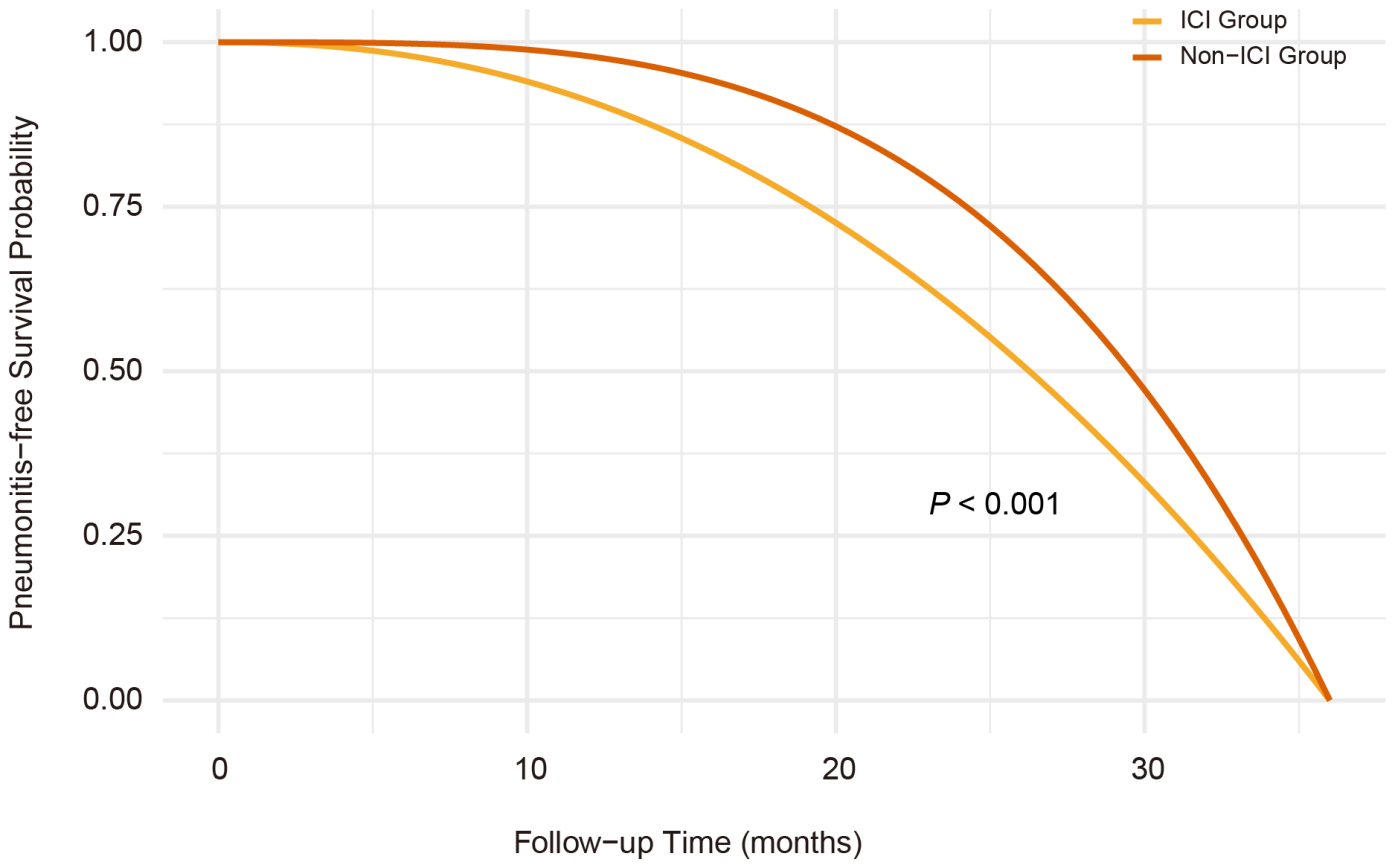
Kaplan-Meier curves of pneumonitis-free survival in radiotherapy-ICI versus radiotherapy-nonICI groups. Pneumonitis-free survival was significantly shorter in the radiotherapy-ICI group than in the radiotherapy-nonICI group (P<0.001, log-rank test), with early divergence of survival curves. The cumulative incidence of pneumonitis was 28.9% in the radiotherapy-ICI group and 10.0% in the radiotherapy-nonICI group.

Over a median follow-up of 4.8 years, pneumonitis occurred in 28.9% of patients receiving RT concurrent with ICIs therapy, compared to 10.0% among non-ICI users. Pneumonitis events accumulated early, with divergence in pneumonitis-free survival emerging within the first few months of therapy, as shown in the time-to-event curves (**Figure 1**). In multivariable Cox regression, RT and ICIs therapy were associated with a significantly elevated risk of pneumonitis (HR 2.86, 95% CI: 2.43–3.29). The risk estimates remained consistent across various adjustment models (**Table 2**).

**Table 2:** Incidence rates and HR for pneumonitis in RT-ICI versus RT-nonICI groups across models and subgroups.

Group	N	Follow-up time (person-years)	No. of pneumonitis	Cumulative incidence (%)	Incidence rate (cases/1000 person-years)	HR (95% CI)
Model 1						
RT-nonICI	12927	22468	1288	9.96	57.33	Reference 2.86 (2.43–3.29)
RT-ICI	8744	15400.3	2523	28.85	163.83	
Model 2						
RT-nonICI	12927	22468	1288	9.96	57.33	Reference 2.86 (2.43–3.29)
RT-ICI	8744	15400.3	2523	28.85	163.83	
Model 3						
RT-nonICI	12927	22468	1288	9.96	57.33	Reference 2.86 (2.43–3.29)
RT-ICI	8744	15400.3	2523	28.85	163.83	
Group						
Autoimmune disease (RA) - No	12805	22240.9	1281	10	57.6	Reference 0.54 (0.46–0.62)
Autoimmune disease (RA) - Yes	1257	227.1	7	5.6	30.83	
Group						
Medication (Antirheumatics) - No	11875	20597.1	1199	10.1	58.21	Reference 0.82 (0.7–0.94)
Medication (Antirheumatics) - Yes	1055	1870.8	89	8.44	47.57	
Group						
Psychiatric comorbidity (Depressive episode) - No	12169	21122.9	1220	10.03	57.76	Reference 0.88 (0.75–1.01)
Psychiatric comorbidity (Depressive episode) - Yes	761	1345.1	68	8.94	50.55	

Incidence of pneumonitis is shown as cumulative incidence (%), incidence rate (per 1,000 person-years), and hazard ratio (HR) with 95% confidence interval (CI) for RT-ICI versus RT-nonICI groups under three statistical models. Subgroup analyses stratified by autoimmune disease (RA), use of antirheumatic medications, and psychiatric comorbidities are also presented.

### 3.3 Risk stratification by subgroup

**Figure 2:**
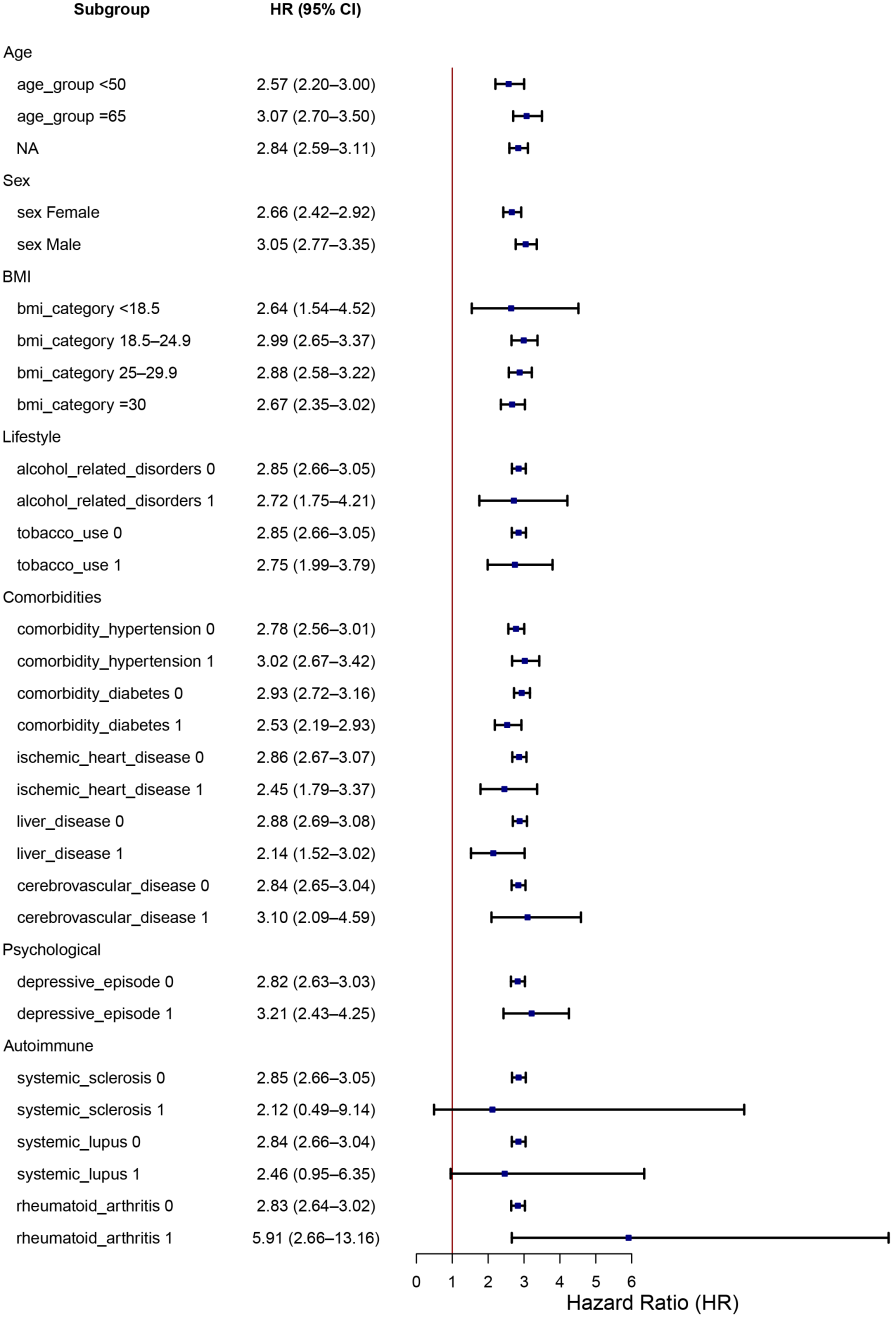
Subgroup analysis of HR for pneumonitis in patients receiving concurrent radiotherapy-ICIs. Forest plot displaying the risk of pneumonitis (hazard ratio and 95% CI) across clinical subgroups including sex, age, BMI, smoking status, comorbid autoimmune disease (RA, SLE), psychiatric conditions, and medication use. The increased risk of pneumonitis remained consistent across most subgroups, with notably higher risk among patients with autoimmune conditions and those using RT-ICI therapy.

In subgroup analyses, the increased risk of pneumonitis associated with RT concurrent with ICIs therapy remained consistent across diverse patient categories, including sex (male, female), age groups (<50, 50–65, ≥65 years), BMI categories (<18.5, 18.5–24.9, 25–29.9, ≥30 kg/m²), and smoking status (non-smokers, smokers). Notably higher risks were observed in patients with pre-existing autoimmune conditions, particularly rheumatoid arthritis (RA), as well as in patients receiving immunomodulatory therapies such as antirheumatic medications. Additionally, psychiatric conditions, specifically depressive episodes, demonstrated a trend toward increased pneumonitis susceptibility, though this association was less pronounced. Detailed subgroup-specific HR and CIs are illustrated in the forest plot (**Figure 2**).

### 3.4 Serum exosomal miRNA signatures in RT-ICI-induced pneumonitis

**Figure 3:**
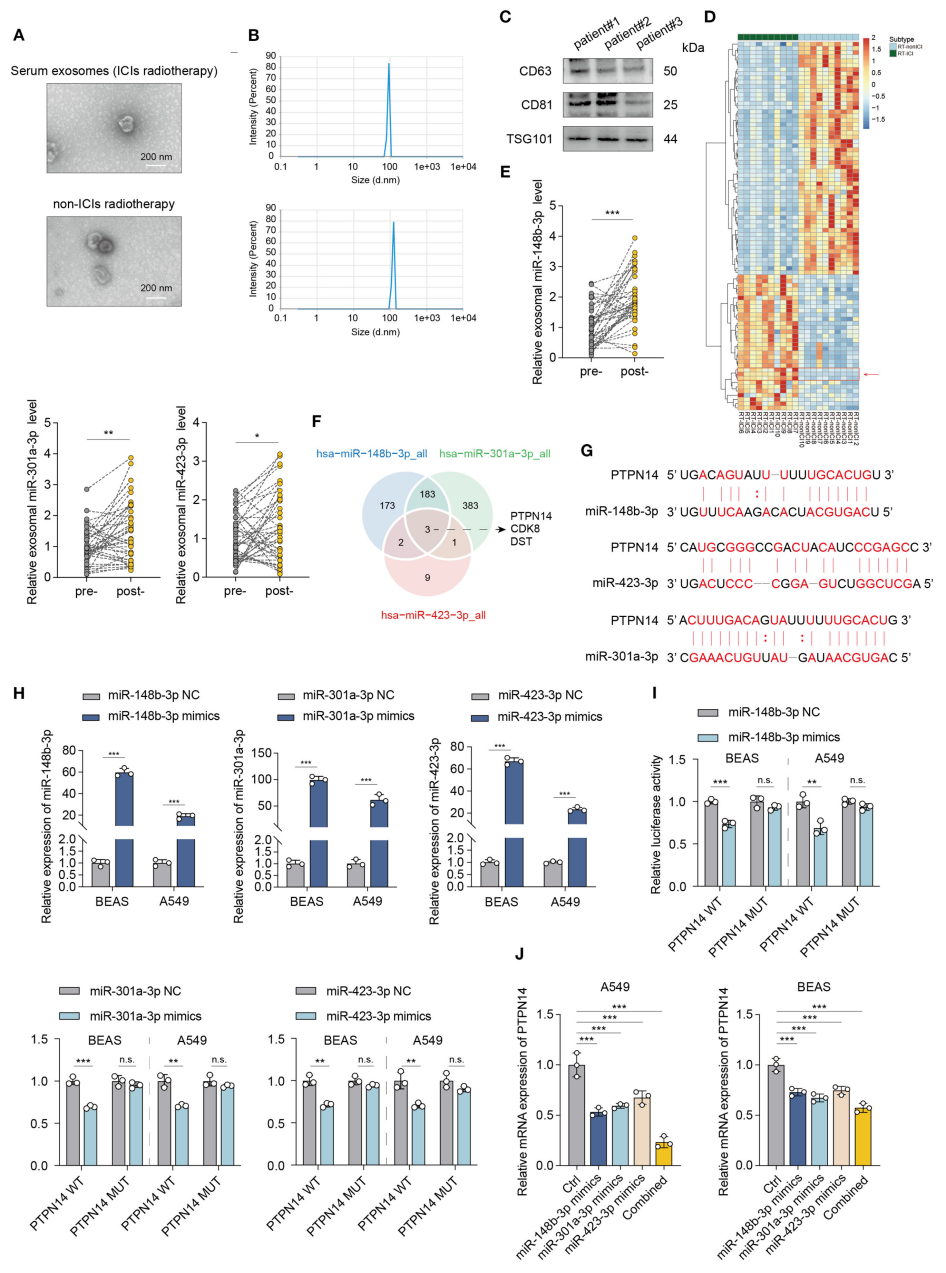
Exosomal miRNA profiling identifies a predictive signature for radiotherapy-ICI-associated pneumonitis. **(A–C)** Characterization of serum exosomes by TEM, NTA, and Western blot (CD81/CD63/TSG101). **(D)** Hierarchical clustering of differentially expressed miRNAs between radiotherapy-ICI and radiotherapy-alone cohorts. **(E)** qPCR analysis of serum miR-148b-3p, miR-301a-3p, and miR-423-3p levels in radiotherapy-ICI patients pre- and post-pneumonitis. **(F)** Venn diagram of target genes shared by the three miRNAs by Starbase database. **(G)** Schematic of miRNA binding sites in the PTPN14 3’UTR. **(H)** qPCR validation of miRNA mimic transfection efficiency. **(I)** Dual-luciferase assay confirming miRNA-PTPN14 3’UTR binding (luciferase activity normalized to WT). **(J)** qPCR analysis of PTPN14 suppression by individual or combined miRNA mimics. *P < 0.05, **P < 0.01, ***P < 0.001.

To identify high-risk subgroups for early preventive intervention, we performed serum exosomal miRNA sequencing on pre-treatment peripheral blood samples from 20 patients who subsequently developed pneumonitis: 10 receiving radiotherapy combined with ICIs and 10 receiving radiotherapy alone. The isolated exosomes exhibited characteristic cup-shaped morphology under TEM, a mean diameter of 100 nm by nanoparticle tracking analysis (NTA), and positive expression of exosomal markers (CD81/CD63/TSG101) by western blot (**Figures 3A–C**). Hierarchical clustering analysis revealed distinct miRNA expression patterns in the radiotherapy-ICI cohort compared to the radiotherapy-alone group (**Figure 3D**). Consequently, we applied LASSO regression to 28 differentially expressed miRNAs and established a predictive model incorporating three miRNAs: miR-148b-3p, miR-301a-3p, and miR-423-3p, with the risk score calculated as: (2.1 × miR-148b-3p) + (1.8 × miR-301a-3p) + (1.1 × miR-423-3p). Serial serum monitoring demonstrated significant post-pneumonitis upregulation of these miRNAs in radiotherapy-ICI patients compared to baseline levels (**Figure 3E**), suggesting their utility in dynamically tracking both tumor response and treatment-related toxicity.

Bioinformatic analysis using the starbase database identified PTPN14, CDK8, and DST as common target genes of these three miRNAs (**Figure 3F**). Notably, tyrosine-protein phosphatase non-receptor type 14 (PTPN14) serves as a crucial tumor and metastasis suppressor that interacts with YAP, the core effector of the Hippo signaling pathway, to block its nuclear translocation, thereby effectively inhibiting YAP’s transcriptional activity and oncogenic functions. As the key downstream effector of the Hippo pathway, the subcellular localization and activity regulation of YAP play pivotal roles in tumorigenesis and progression. As illustrated in the figure, the miR-148b-3p, miR-301a-3p, and miR-423-3p cluster target adjacent sites within the 3’ untranslated region (3’UTR) of PTPN14 mRNA (**Figure 3G**).

To validate the targeting relationship between miR-148b-3p, miR-301a-3p, miR-423-3p and PTPN14, we performed dual-luciferase reporter assays. Following successful transfection with miRNA mimics (as verified by qRT-PCR) (**Figure 3H**), these mimics specifically bound to the PTPN14 3’UTR and significantly suppressed luciferase activity (**Figure 3I**).

Further validation in miRNA-stably transfected cell models showed that transfection with individual miRNA mimics (miR-148b-3p, miR-301a-3p, or miR-423-3p) significantly suppressed PTPN14 expression. Most notably, co-transfection with all three mimics produced the most pronounced inhibitory effect on PTPN14 expression (**Figure 3J**), demonstrating a synergistic effect among these miRNAs in regulating PTPN14.

### 3.5 Intercellular communication analysis

**Figure 4:**
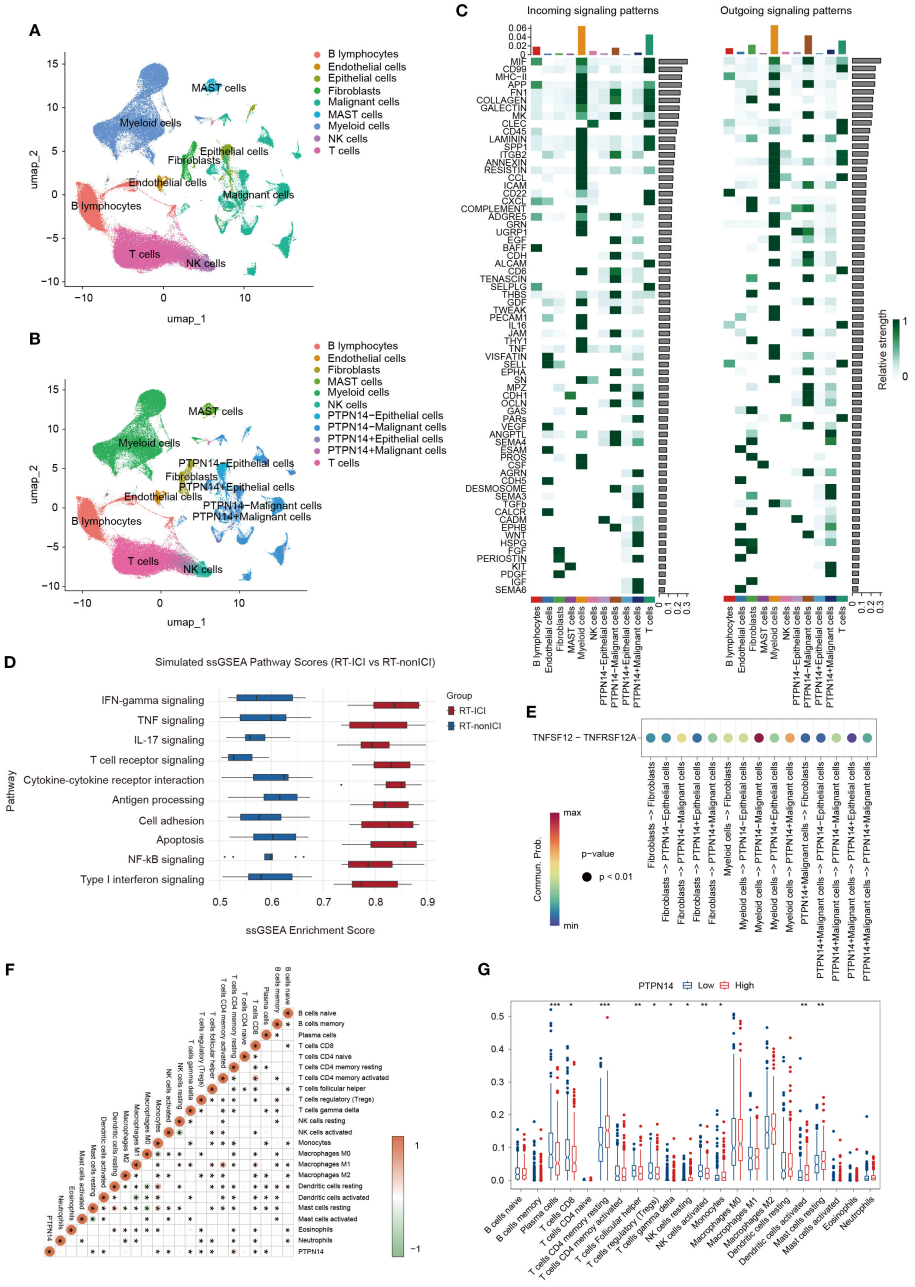
Intercellular communication analysis of lung tissue samples from patients with and without PTPN14 expression. **(A)** UMAP plot identifying various cell types. **(B)** UMAP plot of cell classification based on PTPN14 expression. **(C)** Heatmap of outgoing and incoming signaling between various cell types. **(D)** Dot plot of immune-related enriched pathways, highlighting activation of IFN-γ, Th17, TNF, and JAK-STAT signaling in PTPN14 low samples. **(E)** Ligand-receptor pairs of TNFSF12-TNFRSF12A signaling pathway between various cell types. **(F)** Heatmap of correlation between PTPN14 and various cell types. **(G)** The relative proportions of various cell types in the high and low PTPN14 groups. *P < 0.05, **P < 0.01, ***P < 0.001.

To investigate PTPN14’s role in RT-ICIs pneumonitis, we analyzed scRNA-seq data from GSE131907. Cells were classified into nine types (**Figure 4A**), with a focus on epithelial and malignant cells due to their pneumonitis relevance. These cells were stratified into PTPN14+ and PTPN14- subsets based on zero expression threshold (**Figure 4B**).

The signaling patterns show that PTPN14- malignant cells exhibit stronger interactions with TNF-associated pathways when communicating with other cell types, such as myeloid, fibroblasts cells, and epithelial cells (**Figures 4C, D**). In both malignant and epithelial cells, PTPN14+ subpopulations showed reduced TNFSF12 signaling reception from myeloid cells or fibroblasts compared to PTPN14- cells (**Figure 4E**). This suggests that PTPN14 may modulate TNFSF12-TNFRSF12A-mediated crosstalk between these cell types. Given this pathway’s role in inflammation, cell death, and fibrosis, we propose that PTPN14 attenuates chronic inflammatory signaling, thereby limiting excessive fibrotic remodeling.

CIBERSORT analysis further revealed distinct immune cell profiles between PTPN14-high and PTPN14-low groups. Notably, PTPN14-high samples showed increased resting memory CD4+ T cells (r=0.28, P=1.88E-10) but decreased plasma cells (r=-0.20, P=2.69E-6) compared to PTPN14-low samples (**Figures 4F, G**), suggesting PTPN14 may maintain memory T cells while suppressing plasma cell differentiation.

### 3.6 PTPN14-YAP axis mediates miRNA cluster-induced fibrotic EMT in pneumonitis

**Figure 5:**
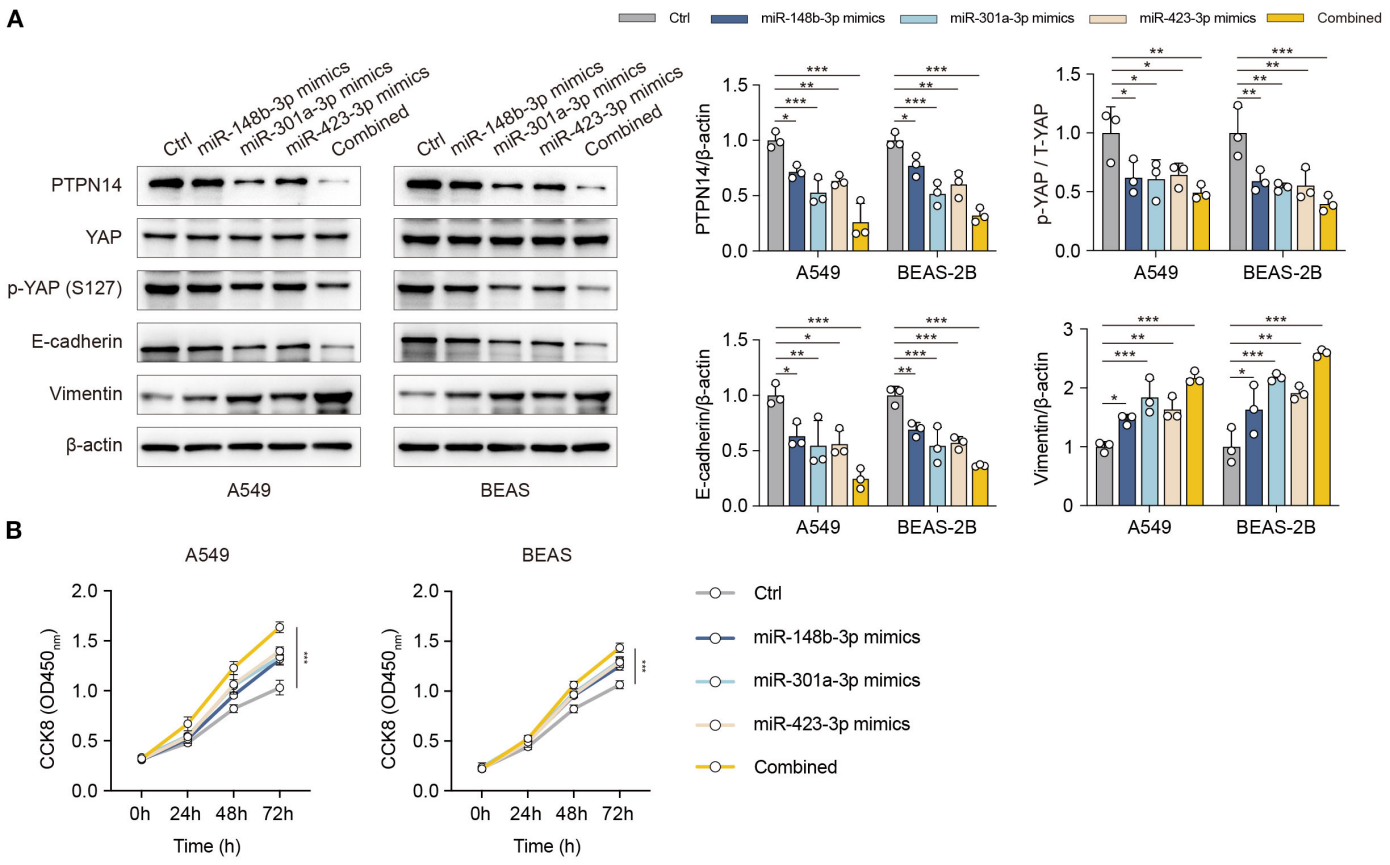
The miR-148b-3p/miR-301a-3p/miR-423-3p cluster promotes EMT-associated fibrosis via the PTPN14/YAP axis. **(A)** Western blot analysis of PTPN14, YAP, p-YAP, E-cadherin, and Vimentin in A549 and BEAS-2B cells following overexpression of the miRNA cluster or individual miRNAs. **(B)** Cell proliferation assessed by CCK-8 assay in cells overexpressing the miRNA cluster or individual miRNAs. Data represent mean ± SD; *P < 0.05, **P < 0.01, ***P < 0.001

Building upon our previous validation that the miR-148b-3p/miR-301a-3p/miR-423-3p cluster directly targets the 3’UTR of PTPN14 mRNA (Section 3.5), we further explored its functional role in promoting epithelial-mesenchymal transition (EMT)-associated fibrosis during pneumonitis. To elucidate the mechanistic link between PTPN14/YAP signaling and EMT progression, we performed comprehensive analyses in human bronchial (BEAS-2B) and alveolar (A549) epithelial cell lines. Overexpression of the miRNA cluster significantly suppressed both PTPN14 and YAP expression, concomitant with characteristic EMT marker alterations (reduced E-cadherin and elevated vimentin levels) (**Figure 5A**). CCK-8 assays demonstrated that co-overexpression of the miR-148b-3p/miR-301a-3p/miR-423-3p cluster significantly suppressed cell proliferation, with greater efficacy than individual miRNA overexpression, further supporting the role of the PTPN14/YAP axis in fibrosis progression (**Figure 5B**). Collectively, our results delineate a novel regulatory axis wherein the miR-148b-3p/miR-301a-3p/miR-423-3p cluster drives YAP-dependent EMT programs through PTPN14 downregulation.

## 4 Discussion

In this large real-world cohort study, we found a significant association between concurrent RT and ICIs and increased risk of pneumonitis (including both radiation pneumonitis and immune-related pneumonitis) in NSCLC. Specifically, we identified a serum exosomal miRNA cluster (miR-148b-3p, miR-301a-3p, miR-423-3p) that predicts this toxicity. The incidence of pneumonitis in the RT-ICI combination group approached 30%, compared to approximately 10% in the radiotherapy receiving non-ICI control group. This rate is substantially higher than those reported in multiple clinical trials, such as KEYNOTE-024 (overall pneumonitis rate of 5.8%, with 2.6% at grade 3 and 4 severity) and CheckMate-057 (pneumonitis rate approximately 4.6%) (21, 22). For instance, an analysis by Shi et al. based on the U.S. FDA Adverse Event Reporting System (FAERS) reported a 4.1% incidence of ICI-related pneumonitis among NSCLC patients, a rate consistent with many clinical trials (23). In addition, multiple meta-analyses have consistently shown that the incidence of pneumonitis among NSCLC patients receiving PD-1/PD-L1 monotherapy typically ranges between 3% and 5% (24). In contrast, the much higher incidence observed in our cohort suggests that the real-world burden of ICI-related pneumonitis may be underestimated in trial-based assessments. Importantly, this elevated risk remained robust across various subgroups and multivariate adjustment models. These findings suggest that the toxicity burden of combination therapy is likely underestimated in controlled trials, emphasizing the need for future research to better represent these clinically relevant but often underrepresented patient groups.

To explore the pathogenesis, we focused on serum exosomal miRNA profiling rather than bulk transcriptomics. We identified a specific miRNA cluster (miR-148b-3p/miR-301a-3p/miR-423-3p) significantly upregulated in patients developing pneumonitis post RT-ICIs. The predictive power of this cluster was consolidated into a risk-score model: (2.1 × miR-148b-3p) + (1.8 × miR-301a-3p) + (1.1 × miR-423-3p). Serial monitoring confirmed significant post-pneumonitis elevation of these miRNAs, supporting their utility for dynamic toxicity tracking. Importantly, this same cluster independently predicted poorer overall survival in NSCLC, underscoring its dual role as a prognostic and toxicity biomarker. Further mechanistic studies revealed that this miRNA cluster promotes pulmonary fibrosis progression by targeting and suppressing PTPN14, activating the YAP/Hippo pathway and inducing EMT. Crucially, overexpressing the miRNA cluster or knockdown PTPN14 exacerbated cell death and fibrosis. Single-cell analyses further revealed that PTPN14 loss disrupts critical TNFSF12-TNFRSF12A-mediated immune crosstalk and promotes an immunosuppressive microenvironment characterized by increased resting CD4+ T cells but decreased plasma cells. Thus, PTPN14 suppression during pneumonitis pathogenesis simultaneously drives fibrosis and compromises anti-tumor immunity.

Despite the strengths of integrating clinical and transcriptomic data, several limitations should be acknowledged. First, although propensity score matching was applied to mitigate confounding, residual bias inherent to observational studies cannot be fully excluded. Second, initial serum exosomal miRNA validation was performed in a limited sample size, warranting validation in larger, multicenter cohorts. Third, pneumonitis diagnosis relied on clinical coding and manual chart review, which may be subject to misclassification or incomplete documentation. Finally, the cross-sectional design precludes correlation of miRNA levels longitudinally with radiographic and functional lung changes.

Nevertheless, this study provides a comprehensive characterization of RT-ICIs-related pneumonitis in NSCLC within a real-world setting, integrating clinical phenotyping with functional genomic profiling. Our findings offer important insights for clinical implementation for risk stratification and lay the foundation for developing individualized predictive models and targeted mitigation strategies. Future prospective, multicenter studies are needed to validate the PTPN14-YAP axis to mitigate toxicity while preserving anti-tumor efficacy.

## 5 Conclusion

In this cohort study, concurrent RT and ICIs significantly increased pneumonitis risk in NSCLC patients, with 28.9% incidence in the RT-ICIs group versus 10.0% in RT-nonICIs controls. This risk was consistent across subgroups, including age, sex, BMI, and comorbidities. Crucially, we identified a serum exosomal miRNA cluster (miR-148b-3p/miR-301a-3p/miR-423-3p) that predicts pneumonitis risk and mechanistically drives fibrosis through PTPN14 suppression-mediated YAP activation. Our study underscores the need for proactive monitoring of this miRNA signature in RT-ICIs therapy patients, particularly those with autoimmune or psychiatric comorbidities, who appear more susceptible to pneumonitis.While consistent with previous studies, the real-world incidence in our cohort was significantly higher than in clinical trials, suggesting that patients excluded from trials may be at greater risk. Thus, real-world data are critical in assessing the risk of RT-ICIs-related pneumonitis.

## Data Availability

The original contributions presented in the study are included in the article/supplementary material. Further inquiries can be directed to the corresponding author.
